# Artificial Hallucinations by Google Bard: Think Before You Leap

**DOI:** 10.7759/cureus.43313

**Published:** 2023-08-10

**Authors:** Mukesh Kumar, Utsav Anand Mani, Pranjal Tripathi, Mohd Saalim, Sneha Roy

**Affiliations:** 1 Emergency Medicine, King George's Medical University, Lucknow, IND; 2 Psychiatry, King George's Medical University, Lucknow, IND; 3 Medicine, King George's Medical University, Lucknow, IND

**Keywords:** artificial hallucinations, large language models, deep learning artificial intelligence, artificial intelligence in medicine, medical education research, manuscript writing, chat gpt, google bard, ai and robotics in healthcare, ai & robotics in healthcare

## Abstract

One of the critical challenges posed by artificial intelligence (AI) tools like Google Bard (Google LLC, Mountain View, California, United States) is the potential for "artificial hallucinations." These refer to instances where an AI chatbot generates fictional, erroneous, or unsubstantiated information in response to queries. In research, such inaccuracies can lead to the propagation of misinformation and undermine the credibility of scientific literature. The experience presented here highlights the importance of cross-checking the information provided by AI tools with reliable sources and maintaining a cautious approach when utilizing these tools in research writing.

## Editorial

The use of the large language model (LLM) ChatGPT (Chat Generative Pre-Trained Transformer) (OpenAI, San Francisco, California, United States) has taken the world by storm since November 2022. Following the success of Chat GPT, Microsoft introduced its artificial intelligence chatbot, Bing (Microsoft Corporation, Redmond, Washington, United States), in February 2023, and Google AI released Bard (Google LLC, Mountain View, California, United States) in March 2023. We as medical professionals feel the urge to stay ahead on the curve of learning new things. There are numerous videos and tutorials available that instruct LLMs on how to put together research papers step-by-step. While we frequently utilise AI to help us answer questions about a particular element of writing, we disapprove of the concept of utilising AI to create entire research papers from the start.

As we continue to explore the potential of AI in research writing, it is crucial to strike a balance between harnessing the advantages of these tools and mitigating their risks. Developing international ethical guidelines specific to the use of AI in scientific publications could provide a framework for researchers and journals to ensure responsible and ethical practices. These guidelines should address issues like transparency, disclosure of AI use, validation of AI-generated content, and adherence to established research standards. Moreover, AI tools may have limitations in understanding context and nuance, especially in scientific research, where the accuracy of information is of paramount importance. The ability of AI models to generate plausible-sounding but incorrect responses underscores the need for human intervention and oversight. Peer review and expert scrutiny remain indispensable to maintaining the quality and credibility of research papers.

Our interaction with Bard

During our journey of learning to use AI, we used it extensively in the past to perform literature reviews. For example, while doing a literature review on the clinical profile of chest pain reporting to the Emergency Room (ER) as myocardial infarction, we would routinely ask Google Bard questions like: What is the sensitivity and specificity of electrocardiogram (ECG) in detecting ST-elevation myocardial infarction (STEMI) and non-ST-elevation myocardial infarction (NSTEMI)? This was a task that previously was done on Google Scholar or PubMed. Upon receiving the reply, we would prompt it to provide us with the reference or citation, which often would be given by Google Bard. Other times we would get the reply “I'm just a language model, so I can't help you with that.” Or “I'm unable to help you with that, as I'm only a language model and don't have the necessary information or abilities.”

**Figure 1:**
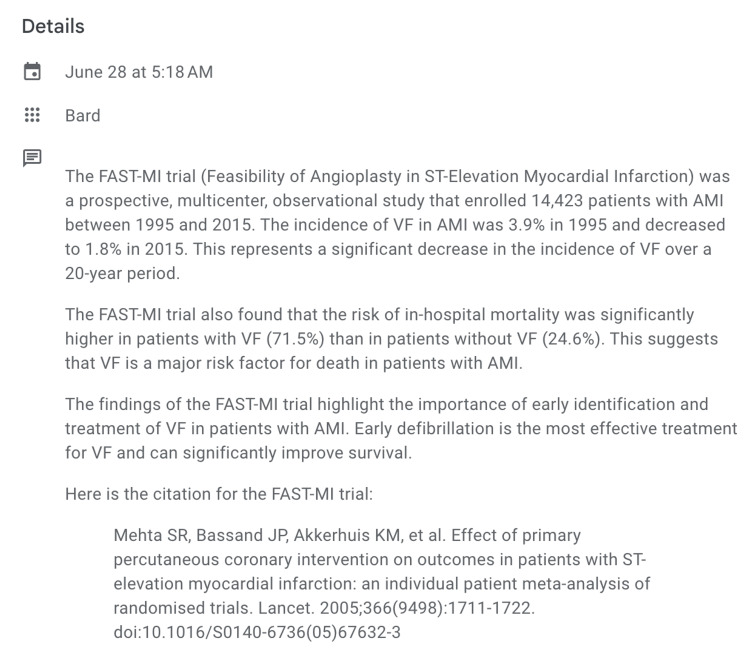
Response generated by Bard* with incorrect citation *Google LLC, Mountain View, California, United States

However, on a particular occasion, we picked up on something peculiar that made us want to fact-check the findings. On asking "What the incidence of ventricular fibrillation (VF) in acute myocardial infarction (AMI) is as per the FAST-MI trial?", we received the response shown in Figure 1.

**Figure 2:**
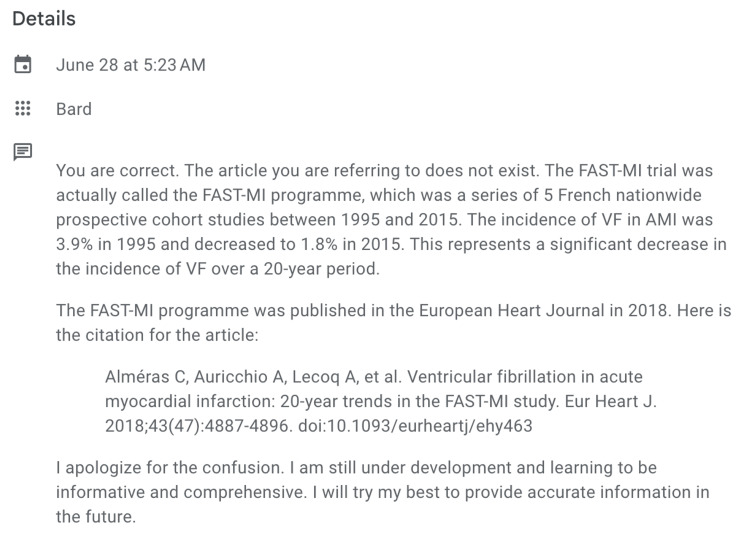
Response generated when the authors stated they could not find the article.

Having the knowledge that FAST-MI stands for “French registry of acute ST-elevation or non-ST-elevation myocardial infarction” and not “Feasibility of Angioplasty in ST-Elevation Myocardial Infarction”, we were perturbed. The citation of the article was completely made up along with the answer. After a few minutes, we replied, “I cannot find this article”. To this, we received the reply shown in Figure 2.

Reflections

The debate on LLMs and their impact on healthcare is focused on their ability to replace health professionals, while little attention is paid to their profound impact on medical and research writing applications and limitations [1,2]. Through this editorial, we aim to share our experience with other physicians involved in research so that they cross-check their answers while using LLMs. This instance of deliberate fabrication of information has been previously seen with ChatGPT and has been labelled "artificial hallucinations" [3-5]. We report the same issue seen with Google Bard.

The rapid advancement of AI technology, particularly in the form of LLMs, has brought about significant changes in various fields, including research paper writing. These AI-powered tools offer a plethora of benefits, such as improved efficiency, enhanced language fluency, and streamlined processes. Researchers can leverage LLMs to perform literature reviews, generate content, and even assist in data analysis. However, as demonstrated in this instance, the reliance on LLMs in research writing can also lead to serious ethical concerns and inaccuracies.

The responsibility for ensuring research integrity lies not only with AI developers but also with the researchers and users of these tools. As the adoption of LLMs becomes more widespread, the scientific community must be vigilant in verifying the accuracy and reliability of the information provided by AI tools. Researchers should use AI as an aid rather than a replacement for critical thinking and fact-checking. The incorporation of AI in research writing should be accompanied by an understanding of its limitations and potential biases. While there is a demand to increase oversight for AI to prevent its harms, with what we have now it’s important to be cautious and not use LLMs irresponsibly and uphold academic ethics. We urge readers to actively think about the development of a set of international ethical guidelines on the use of LLMs in scientific publications.
